# On the absence of a phonon bottleneck in strongly confined CsPbBr_3_ perovskite nanocrystals†
†Dedicated to Dalian Institute of Chemical Physics, CAS, on the occasion of its 70th anniversary.
‡
‡Electronic supplementary information (ESI) available: Fig. S1–S13 and wavefunction calculations. See DOI: 10.1039/c9sc01339c


**DOI:** 10.1039/c9sc01339c

**Published:** 2019-05-06

**Authors:** Yulu Li, Runchen Lai, Xiao Luo, Xue Liu, Tao Ding, Xin Lu, Kaifeng Wu

**Affiliations:** a State Key Laboratory of Molecular Reaction Dynamics , Dynamics Research Center for Energy and Environmental Materials , Dalian Institute of Chemical Physics , Chinese Academy of Sciences , Dalian , 116023 , China . Email: kwu@dicp.ac.cn; b Departmental of Chemistry , College of Chemistry and Chemical Engineering , Xiamen University , Xiamen , Fujian 361005 , China

## Abstract

In traditional solar cells, photogenerated energetic carriers (so-called hot carriers) rapidly relax to band edges *via* emission of phonons, prohibiting the extraction of their excess energy above the band gap. Quantum confined semiconductor nanocrystals, or quantum dots (QDs), were predicted to have long-lived hot carriers enabled by a phonon bottleneck, *i.e.*, the large inter-level spacings in QDs should result in inefficient phonon emissions. Here we study the effect of quantum confinement on hot carrier/exciton lifetime in lead halide perovskite nanocrystals. We synthesized a series of strongly confined CsPbBr_3_ nanocrystals with edge lengths down to 2.6 nm, the smallest reported to date, and studied their hot exciton relaxation using ultrafast spectroscopy. We observed sub-ps hot exciton lifetimes in all the samples with edge lengths within 2.6–6.2 nm and thus the absence of a phonon bottleneck. Their well-resolved excitonic peaks allowed us to quantify hot carrier/exciton energy loss rates which increased with decreasing NC sizes. This behavior can be well reproduced by a nonadiabatic transition mechanism between excitonic states induced by coupling to surface ligands.

## Abstract 2

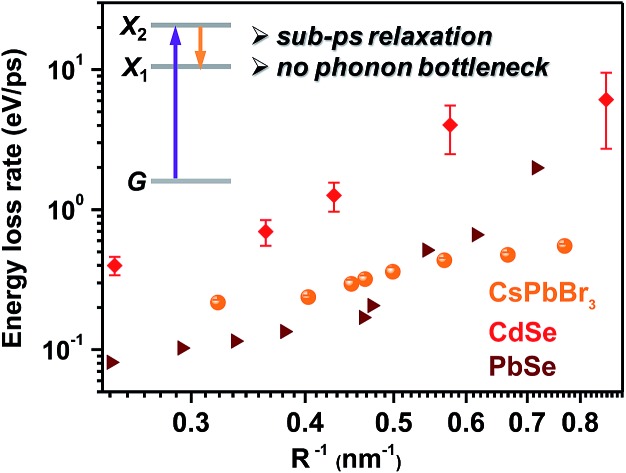
Strongly confined CsPbBr_3_ perovskite nanocrystals exhibit sub-ps hot exciton relaxation dynamics and the absence of a phonon bottleneck.

## Introduction

In typical semiconductors, photogenerated carriers with excess energy above band edges, the so-called hot carriers, rapidly relax to band edges mainly *via* emission of phonons. The relaxation often occurs on a sub-ps timescale,^1^–^4^ making the excess energy of hot carriers very difficult to harvest. As a result, this part of energy is wasted as heat in conventional optoelectronic devices such as solar cells, which is a major reason for the Shockley–Queisser limit for single-junction solar cells.^5^ If hot carriers can be efficiently extracted to selective contacts,^6^–^8^ the efficiency of solar cells can be pushed to as high as 66%.^6^,^9^ As such, it is essential to understand the mechanisms of hot carrier relaxation and to prolong hot carrier lifetimes for next-generation solar cells.

Lead halide perovskites (APbX_3_; with A = Cs, MA, and FA, and X = Cl, Br, I) have recently been proven to be technologically important for many light-harvesting and -emitting applications.^10^–^12^ Hot carrier dynamics in these materials have also been intensively studied because of recent reports of long-lived hot carriers.^13^–^15^ It was proposed that their highly dynamic perovskite lattices facilitated the formation of large polarons which not only protected band edge carriers from trapping but also slowed down hot carrier cooling if the rate of polaron formation was faster than that of hot carrier relaxation.^13^,^16^ On the other hand, however, recent studies indicated sub-ps hot carrier cooling in various types of bulk-like lead halide nanocrystals (NCs) including CsPbX_3_ NCs,^17^–^20^ MAPbBr_3_ NCs,^19^,^21^ and FAPbBr_3_ NCs^19^,^22^ at low excitation energy densities. Note that slower carrier cooling on 10s of ps timescale reported in ref. 19–22 is a result of Auger heating and *hot* phonon bottlenecks that are significant only at higher excitation densities, which is beyond the scope of this work.

In light of these previous studies, it is interesting to examine whether strong quantum confinement can enable a phonon bottleneck and hence slow down hot carrier relaxation in lead halide perovskites, particularly because the Auger-type, electron-to-hole energy transfer mechanism responsible for sub-ps hot electron relaxation in typical CdSe quantum dots (QDs)^23^–^25^ should be unavailable in these perovskite materials with similar electron and hole effective masses. This phonon bottleneck was theoretically predicted in a very recent study which calculated hot carrier cooling in CsPbBr_3_ NCs using the Red-field theory.^26^ Experimentally, however, the effect of quantum confinement on hot carrier/exciton relaxation in perovskite NCs has never been systematically studied. Such a systematic study was mainly hindered by the lack of methods to synthesize mono-disperse and strongly confined perovskite NCs due to their rapid growth rates in hot-injection synthesis. Recent progress has enabled controllable synthesis of high-quality CsPbBr_3_ NCs in the quantum confinement regime,^27^–^29^ which should allow for examination of the effect of quantum confinement on hot carrier/exciton cooling as has been done for CdSe and PbSe QDs.^23^,^24^,^30^–^32^

Here we study hot exciton relaxation dynamics in mono-disperse CsPbBr_3_ NCs with varying edge lengths (*L*) in the range of 2.6 to 6.2 nm using transient absorption (TA) spectroscopy. We found the absence of a phonon bottleneck. The hot carrier/exciton energy loss rates increased with decreasing NC sizes. Similar electron and hole densities of states (near the band edge) in CsPbBr_3_ exclude the Auger type electron-to-hole energy transfer mechanism. Temperature-independent relaxation rates were inconsistent with the nonadiabatic multi-phonon emission mechanism. NCs capped with alkane thiol ligands of low infrared absorbance showed similar relaxation rates to pristine NCs capped with oleic acid, which also excluded the mechanism of energy transfer to ligands. The hot exciton relaxation mechanism in CsPbBr_3_ NCs is most likely associated with nonadiabatic coupling between excitonic states and surface ligands.

## Results and discussion

### Size-dependent optical properties of CsPbBr_3_ NCs

**Fig. 1:**
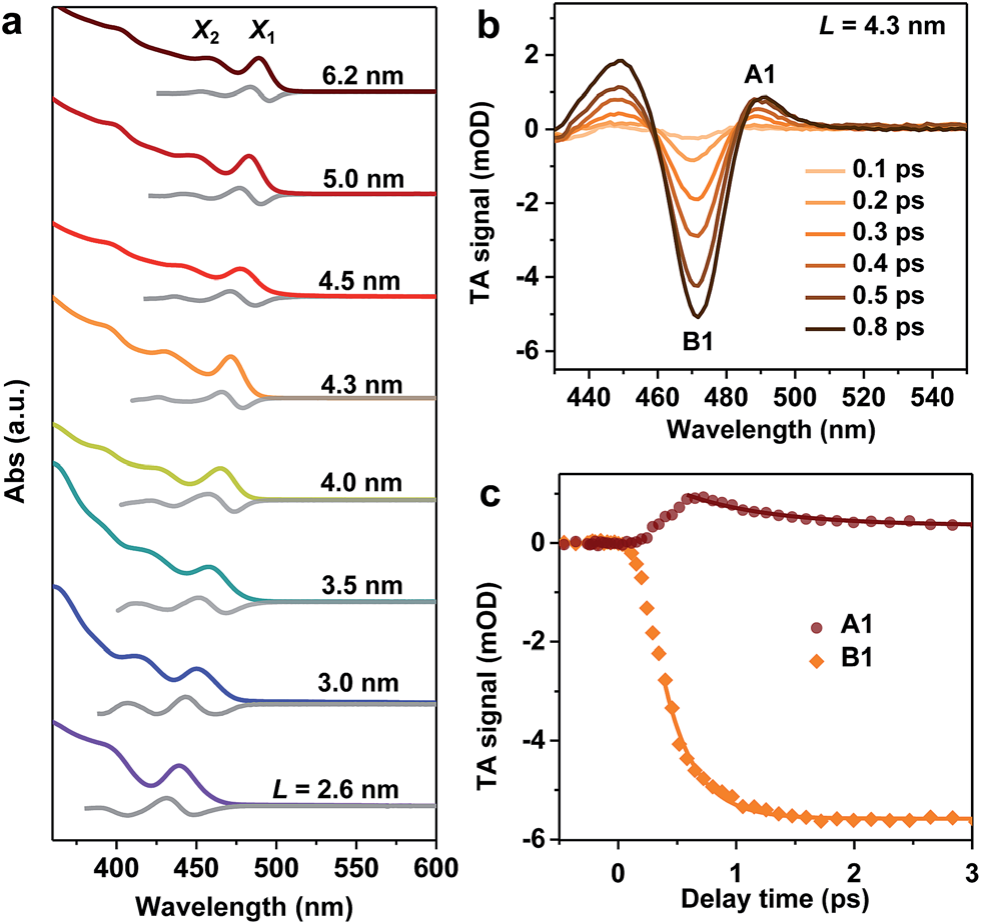
(a) Absorption (colored lines) and first derivative of absorption (gray lines) spectra of NCs of varying sizes. The positions of X_1_ and X_2_ on these spectra are labelled. (b) Transient absorption (TA) spectra of *L* = 4.3 nm NCs at indicated delays following the excitation with a 430 nm pulse. The bleach feature B1 and absorptive feature A1 are labelled. (c) TA kinetics probed at the peaks of A1 (wine circles) and B1 (orange diamonds) are their single-exponential fits (solid lines).

We synthesized mono-disperse CsPbBr_3_ NCs with varying sizes by modifying recent literature methods;^29^,^33^ details can be found in the Methods section. Transmission Electron Microscopy (TEM) images of these NCs are presented in Fig. S1 in the ESI.‡ The edge lengths (*L*) of these cube-shaped NCs are in the range of 2.6 to 6.2 nm (Table 1), which are smaller than the Bohr exciton diameter of CsPbBr_3_ (∼7 nm (ref. 28)), and hence, all samples fall in the strong quantum confinement regime.^34^ Due to the relatively precise control of their sizes, these NCs exhibit well-defined excitonic peaks in their absorption spectra (Fig. 1a). In particular, the two lowest energy peaks can be well-resolved, which are labelled X_1_ and X_2_. According to a recent study,^35^ X_1_ and X_2_ can be assigned to transitions from the first and second hole levels in the valence band to the first and second electron levels in the conduction band, respectively. The exact peak positions for X_1_ and X_2_ were determined from the zero-crossing points on the first derivative curves of absorption spectra (Fig. 1a). Particularly noteworthy is the *L* = 2.6 nm sample, which is the smallest size of CsPbBr_3_ NCs reported to date and thus has the strongest quantum confinement (with X_1_ at ∼439 nm). In previous studies, the smallest size reported was ∼3.0 nm (with X_1_ at ∼451 nm).^28^ The energy separations between X_1_ and X_2_ (Δ*E*) in the samples are in the range of 0.18 eV to 0.32 eV, increasing with decreasing NC sizes (Table 1).

**Table 1:** Summary of sample information for CsPbBr_3_ NCs

*L* (nm)	X_1_ (eV)	X_2_ (eV)	Δ*E* (eV)	*τ*_r_ (fs)	d*E*/d*t* (eV ps^–1^)
6.2 ± 0.3	2.54	2.72	0.18	420 ± 20	0.22 ± 0.01
5.0 ± 0.4	2.57	2.79	0.22	400 ± 10	0.24 ± 0.01
4.5 ± 0.2	2.60	2.83	0.23	390 ± 30	0.30 ± 0.02
4.3 ± 0.2	2.62	2.88	0.26	410 ± 30	0.32 ± 0.02
4.0 ± 0.4	2.67	2.94	0.27	380 ± 30	0.36 ± 0.03
3.5 ± 0.3	2.71	3.03	0.32	370 ± 20	0.44 ± 0.02
3.0 ± 0.2	2.75	3.08	0.33	350 ± 10	0.48 ± 0.02
2.6 ± 0.3	2.82	3.19	0.37	340 ± 30	0.55 ± 0.02

Well-defined excitonic peaks enable the study of state-to-state hot carrier/exciton relaxation dynamics in CsPbBr_3_ NCs using pump–probe TA spectroscopy; see the Methods section for details. Fig. 1b shows the TA spectra of *L* = 4.3 nm NCs within 1 ps following the excitation by a 430 nm pump pulse which selectively excites X_2_. The excitation energy densities were maintained low enough in all TA experiments such that single exciton dynamics were detected.^36^ In the examined time window, TA spectra are dominated by an exciton bleach (B1) feature at ∼470 nm and an induced absorption (A1) at ∼490 nm. B1 can be assigned to the state-filling effect by band edge excitons (both electrons and holes),^37^,^38^ whereas A1 is often attributed to Stark-effect-like signals arising from interactions between hot and band edge excitons.^17^,^39^–^41^ As a result, in the course of hot exciton relaxation to form band edge excitons, the growth of B1 is accompanied by the decay of A1, as observed in Fig. 1c. By simultaneously fitting the kinetics probed at these two features, the X_2_ to X_1_ relaxation time constant is determined to be *τ*_r_ = 410 ± 30 fs. This fast relaxation is similar to that previously reported for bulk-like CsPbBr_3_ NCs,^17^–^20^ indicating that quantum confinement does not slow down hot exciton relaxation in these NCs and that there are relaxation mechanisms bypassing the phonon bottleneck which, however, are overlooked in the calculations in ref. 26. The hot carrier energy loss rate (d*E*/d*t*) can be estimated as Δ*E*/2*τ*_r_, with the factor 2 arising from the assumption of symmetric electron and hole confinement energies, and is 0.32 ± 0.02 eV ps^–1^ for this *L* = 4.3 nm sample. We calculated hot carrier instead of hot exciton energy loss rates simply because previous studies on CdSe^23^ and PbSe^30^ QDs reported hot carrier energy loss rates; see discussions below.

We notice that excitation at 400 nm generates similar hot carrier relaxation dynamics to the case of 430 nm excitation (Fig. S2‡), suggesting that the relaxation from X_*n*_ (*n* ≥ 3) to X_2_ occurs at a much faster rate than that from X_2_ to X_1_. Thus, the relaxation from X_2_ to X_1_ is the rate determining step (RDS) in the complicated multi-step hot exciton relaxation process. Similar observations were found for other samples, such as the *L* = 4.0 nm and *L* = 4.5 nm NCs shown in Fig. S2.‡ It also justifies previous reports in which the same excitation wavelength such as 400 nm was used to determine hot exciton relaxation times in samples with different band edge exciton energy.^17^,^18^

**Fig. 2:**
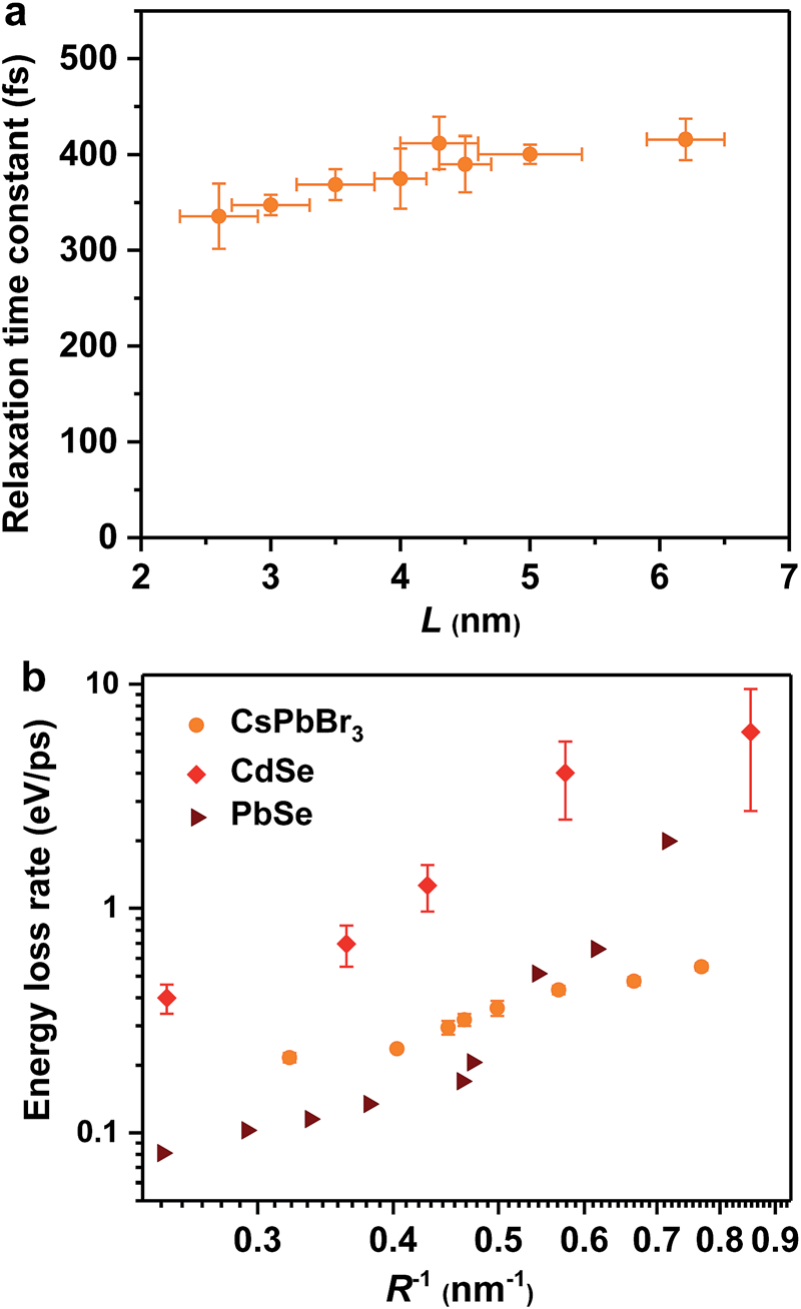
Hot exciton relaxation time constants (a) and hot carrier energy loss rates (b) of CsPbBr_3_ NCs as functions of NC size (orange circles). Note that in order to compare cube-like CsPbBr_3_ NCs with previously reported spherical QDs in (b), we define *R* = *L*/2 for these NCs. The results for CdSe QDs (red diamonds) and PbSe QDs (wine triangles) are adapted from ref. 23 and 30, respectively.

Hot exciton relaxation dynamics in CsPbBr_3_ NCs of other sizes were also measured using TA (see Fig. S3–S9‡) to extract their hot exciton relaxation time constants (*τ*_r_) and hot carrier energy loss rates (d*E*/d*t*) using the procedures described above. The relaxation time constants are in the range of 340–420 fs and depend very weakly on NC sizes (Table 1 and Fig. 2a). In contrast, hot carrier energy loss rates increase with decreasing NC sizes (Table 1 and Fig. 2b). The hot carrier energy loss rates for CdSe and PbSe QDs adapted from ref. 23 and 30, respectively, are also plotted in the figure for comparison. Note that in order to compare cube-like CsPbBr_3_ NCs with previously reported spherical QDs, we define *R* = *L*/2 for these NCs. In general, the hot carrier energy loss rate in CsPbBr_3_ NCs is slower than that in CdSe QDs of comparable sizes. In contrast, there exists a cross-over between CsPbBr_3_ NCs and PbSe QDs; the rate is slightly faster in CsPbBr_3_ NCs for *R* < 2 nm whereas it becomes faster in PbSe QDs for *R* > 2 nm. Possible reasons for these behaviors are given below.

### Temperature and ligand independent relaxation dynamics

We examine possible mechanisms for the ultrafast hot exciton relaxation dynamics observed in CsPbBr_3_ NCs. As we mentioned in the Introduction, hot electron relaxation in II–VI group QDs (such as CdSe) has been attributed to the Auger-type, electron-to-hole energy transfer mechanism,^24^,^25^ because holes in these QDs can quickly relax *via* emission of phonons due to the high density of states in the valence band.^34^ This mechanism should not be applicable for CsPbBr_3_ NCs. Because of similar conduction and valence band structures near their edges and electron and hole effective masses,^12^ both electron and hole energy levels in quantum-confined CsPbBr_3_ NCs should be discrete. This situation is similar to IV–VI group QDs (such as PbSe). The absence of the Auger relaxation mechanism might be responsible for the slower hot carrier energy loss rates of CsPbBr_3_ NCs and PbSe QDs than that of CdSe QDs plotted in Fig. 2b. Still, the energy loss rates of CsPbBr_3_ NCs and PbSe QDs are fast, only several fold slower than that of CdSe, suggesting alternative hot carrier/exciton cooling mechanisms in CsPbBr_3_ and PbSe.

**Fig. 3:**
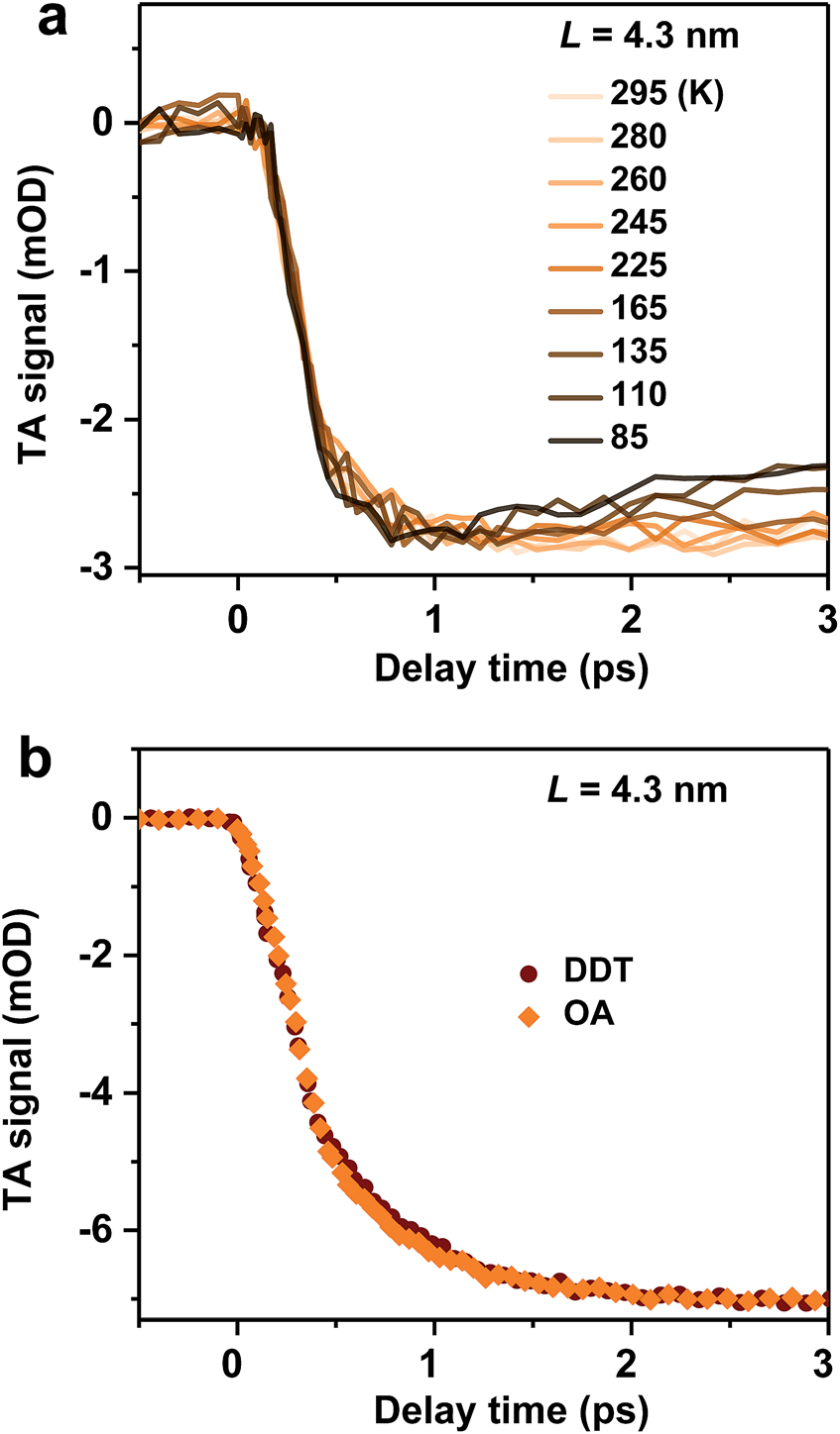
(a) Hot exciton relaxation dynamics (monitored at B1) for *L* = 4.3 nm NCs at varying temperatures. (b) Hot exciton relaxation dynamics (monitored at B1) for *L* = 4.3 nm pristine NCs (with OA ligands; orange diamonds) and DDT-capped NCs (wine circles).

For PbSe QDs, a multi-phonon emission mechanism was proposed to account for the ultrafast hot carrier/exciton relaxation.^30^ This mechanism is enabled by intrinsic nonadiabatic interactions induced by electron–lattice coupling and it requires thermal activation to cross the intersection point between 1*P* and 1*S* potential energy surfaces. Fig. 3a shows the hot exciton relaxation dynamics (by monitoring B1 formation) for *L* = 4.3 nm NCs measured at varying temperatures. The kinetic traces are virtually the same within the noise level, showing negligible temperature dependence. Measurements on another sample (*L* = 4.0 nm) show similar results (Fig. S10‡). Thus, we can exclude the nonadiabatic multi-phonon emission as a major mechanism for hot exciton relaxation in CsPbBr_3_ NCs.

In addition to intrinsic processes inside the NC volume, organic ligands on NC surfaces also act as a pathway for energy dissipation *via* nonradiative, long-range electronic-to-vibrational energy transfer.^42^,^43^ For example, the 1*P* to 1*S* relaxation rate in II–VI core/shell QDs was found to be strongly influenced by surface ligands;^44^ the relaxation rate can be limited by more than one order of magnitude when the capping ligands were changed from stearic acid to 1-dodecanethiol (DDT) because the latter has a much weaker absorbance in the range of ∼0.2–0.3 eV. In order to test this possibility for CsPbBr_3_ NCs, we replaced their pristine oleic acid (OA) ligands with DDT using a literature method (see Methods and Fig. S11‡ for details). As shown in Fig. 3b, OA and DDT capped NCs (*L* = 4.3 nm) show similar B1 formation dynamics. Measurements on other two samples (*L* = 4.0 and 5.0 nm) show similar results (Fig. S12‡). Because these three samples have X_2_ and X_1_ energy differences (Δ*E*) exactly in the range of 0.2–0.3 eV (Table 1), similar ultrafast relaxation rates observed for OA and DDT capped NCs suggest that energy transfer to ligands is not the major channel for hot exciton relaxation either.

### Hot exciton relaxation *via* nonadiabatic interactions

In addition to the electron-to-hole energy transfer, multi-phonon emission and electron-to-ligand energy transfer mechanisms mentioned above, a more complete and unified picture for hot carrier relaxation was established by Kambhampati *et al.* by performing more advanced state-resolved, pump–probe spectroscopic studies on QDs.^40^,^41^ This picture incorporates yet another important relaxation mechanism which is unique for these quantum-confined nanoscale systems – nonadiabatic transition between excitonic states induced by surface ligands.^31^,^32^ This mechanism was invoked to explain the absence of a phonon bottleneck for hot hole relaxation in CdSe QDs.^31^,^32^ Specifically, while hot electron relaxation in CdSe QDs occurs *via* energy transfer to the holes, hot hole relaxation was expected to display a phonon bottleneck. Experimentally, however, hot hole relaxation rates are similarly fast for CdSe QDs with various sizes,^31^,^32^ similar to our observations here for CsPbBr_3_ NCs. In order to account for this peculiar behavior, Cooney *et al.* proposed the nonadiabatic relaxation channel mediated by surface ligands.^31^,^32^ According to this mechanism, nonadiabatic hot carrier relaxation rates (1/*τ*_r_) scale with the Hellman–Feynman force describing electron–nuclear interactions inducing the nonadiabatic transition (*H*) and inversely with the energy gap (Δ*E*): 1/*τ*_r_ ∝ *H*/Δ*E*. As both *H* and Δ*E* increase with decreasing QD sizes, the hot carrier relaxation rates (1/*τ*_r_) are relatively size-independent. On the other hand, the energy loss rates should scale with *H*: d*E*/d*t* = Δ*E*/2*τ*_r_ ∝ *H*.

**Fig. 4:**
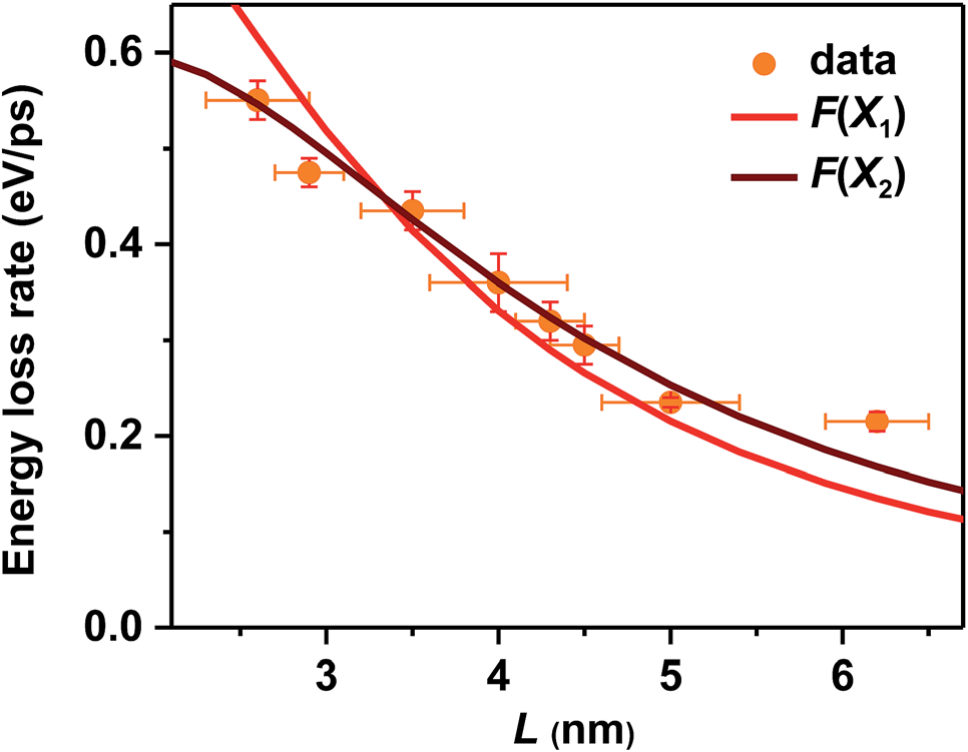
Measured hot carrier energy loss rates (orange circles) and calculated wavefunction surface fraction (*F*) of X_1_ (red solid line) and X_2_ (wine solid line) for CsPbBr_3_ NCs of varying sizes. *F*(X_1_) and *F*(X_2_) have been appropriately scaled by constant factors.

For surface ligand induced nonadiabatic relaxation, *H* should be proportional to the carrier/exciton wavefunction near NC surfaces, which increases with decreasing NC sizes. Following the procedures in ref. 31, we calculated wavefunctions for X_1_ and X_2_ using effective mass approximation (EMA) and obtained their surface fraction (*F*) by integrating wavefunctions in the outermost unit cells and those tunneling into ligand shells; see the ESI‡ for details. It is the surface fraction that interacts effectively with the ligand vibrational modes, and induces nonadiabatic transition from X_2_ to X_1_. The calculated surface fractions (*F*) for X_1_ and X_2_ are plotted in Fig. S13.‡ As shown in Fig. 4, experimental energy loss rates (d*E*/d*t*) can be well fitted using: d*E*/d*t* = *CF*, with *C* being a scaling constant. Because *F*(X_1_) and *F*(X_2_) show similar size-dependence, both can be used to fit the data. This fit provides strong evidence that hot carrier/exciton relaxation in CsPbBr_3_ NCs is mainly enabled by nonadiabatic transition induced by surface ligands. Better agreement with experimental data might be achieved by accounting for minor contributions from other factors such as phonon emission which becomes more important for larger-size NCs with smaller Δ*E*.

### Discussion

In principle, hot carrier/exciton relaxation *via* nonadiabatic transition induced by surface ligands should be universal for quantum-confined colloidal NCs. As such, this channel is also active for CdSe and PbSe QDs. For CdSe QDs, however, the Auger-type, electron-to-hole energy transfer adds an extra and dominant channel for hot electron relaxation, which explains their faster energy loss rates than PbSe QDs and CsPbBr_3_ NCs. PbSe QDs have similar but slightly lower hot carrier energy loss rates than CsPbBr_3_ NCs for *R* > 2 nm in which regime both are likely dominated by the ligand-induced nonadiabatic mechanism. However, PbSe QDs have yet another lattice-induced nonadiabatic mechanism which is also strongly enhanced by quantum confinement; as a result, their hot carrier energy loss rates become faster than those of CsPbBr_3_ NCs when *R* < 2 nm. These explanations qualitatively rationalize the comparison shown in Fig. 2b. Nonetheless, several unknowns remain to be elucidated for a more quantitative interpretation of Fig. 2b. The first one is related to how to quantify the ligand-induced nonadiabatic relaxation rates for NCs of different compositions. This would explain the slightly slower hot carrier energy loss rates in PbSe QDs than in CsPbBr_3_ NCs for *R* > 2 nm. Another unknown is why the lattice-induced nonadiabatic mechanism is significant in PbSe QDs but not in CdSe QDs and CsPbBr_3_ NCs.

A more general issue associated with the study of hot carrier dynamics of colloidal NCs is how to disentangle the various hot carrier relaxation mechanisms and how to determine the absolute rate for each individual pathway. According to the unified relaxation picture established by Kambhampati *et al.*,^40^,^41^ in many cases these mechanisms co-exist and thus it is very difficult to cleanly separate them. Experimental studies with more extensively tuned relaxation-related parameters (NC size, electronic structure, temperature, *etc.*) combined with high-level theories would be required for this goal, which is beyond the scope of this work. The focus here is to unravel the dominant hot carrier relaxation mechanism responsible for the absence of a phonon bottleneck in strongly confined perovskite NCs.

## Conclusions

In summary, we measured size-dependent hot exciton relaxation dynamics in mono-disperse and strongly quantum-confined CsPbBr_3_ NCs using TA spectroscopy and found the absence of a phonon bottleneck. Their well-resolved excitonic peaks allowed us to calculate size-dependent energy loss rates. The energy loss mechanism for CsPbBr_3_ NCs is inconsistent with that proposed for both CdSe (electron-to-hole energy transfer) and PbSe QDs (nonadiabatic multi-phonon emission). Energy transfer to surface ligands is not the dominant mechanism either, because NCs capped with ligands of low and high infrared absorbances at the X_2_ to X_1_ transition energy showed essentially the same relaxation dynamics. Rather, hot exciton relaxation in strongly confined CsPbBr_3_ NCs is likely dominated by a nonadiabatic transition mechanism induced by surface ligands. Smaller NCs have stronger quantum confinement and thus higher wavefunction amplitude at NC surfaces, thus enhancing their interaction with the nuclear coordinates of surface ligands and bypassing the otherwise expected phonon bottleneck. This study highlights the difficulty of achieving long-lived hot carriers even in strongly confined perovskite NCs, due to the ligand-induced nonadiabatic transition mechanism that universally exists for colloidal NCs.

## Methods

### Synthesis of CsPbBr_3_ NCs

We synthesized CsPbBr_3_ NCs by modifying previously reported procedures.^29^ The synthesis started with the preparation of Cs oleate precursors. 0.25 g Cs_2_CO_3_, 0.8 g oleic acid (OA), and 7 g 1-octadecene (ODE) were loaded into a 50 mL 3-neck flask and vacuum-dried for 1 h at 120 °C using a Schlenk line. The mixture was heated under an argon atmosphere to 150 °C until all the Cs_2_CO_3_ was dissolved. The Cs-oleate precursor solution was kept at 100 °C to prevent precipitation of Cs-oleate out of ODE. In another 25 mL 3-neck flask, a precursor solution of Pb and Br was prepared by dissolving 75 mg PbBr_2_ and varying amounts of ZnBr_2_ (0–600 mg) in a mixture of ODE (5 mL), OA (3 mL), and oleylamine (OAm, 3 mL). After the precursor solution of Pb and Br was vacuum-dried for 1 h at 120 °C, it was set to the reaction temperature under an argon atmosphere. The reaction temperature was varied depending on the desired NC sizes (88 °C for 2.6 nm, 93 °C for 3.0 nm, 100 °C for 3.5 nm, 110 °C for 4.0 nm, 120 °C for 4.3 nm and 4.5 nm, 130 °C for 5.0 nm and 140 °C for 6.2 nm QDs). When the reaction temperature was reached, 0.4 mL of Cs precursor solution was swiftly injected to initiate the reaction. The reaction was quenched after a short period of time (10–180 s, depending on the temperature in the range of 190–80 °C) by cooling the flask in an ice bath. After the crude solution was cooled down to room temperature, the product was centrifuged at 3500 rpm for 15 min to remove the unreacted salts as the precipitate, and the NCs dispersed in the supernatant were collected. For reactions conducted at higher temperatures (140–190 °C), only small amounts of unreacted salts remained in the solution, resulting in a higher reaction yield. 8 mL of acetone was directly added to the supernatant to precipitate the NCs followed by centrifugation at 3500 rpm for 3 min. For reactions conducted at lower temperatures (80–120 °C), larger amounts of unreacted salt remained in the supernatant after centrifugation. In this case, the supernatant was left on the benchtop under ambient conditions for ∼0.5 hour until the salts precipitated. Then the mixture was centrifuged at 3500 rpm for another 15 min. After obtaining a clear supernatant, the NCs were precipitated by dropwise adding acetone until the mixture just turned turbid to avoid decomposition of the NCs. Then the dried NCs were collected and dissolved in hexane. Note that in order to obtain very small size NCs (2.6–3.5 nm), the synthesis was scaled up by 3 times, and the reaction should be performed for relatively longer times (180 s for 2.6 nm, 120 s for 3.0 nm, and 100 s for 3.5 nm) without obvious temperature fluctuations.

### Thiol ligand exchange

The thiol ligand exchange followed previously reported procedures.^45^ 2 mL 1-dodecanethiol (DDT) and 5 mL hexane were vacuum-dried for 1 h at 100 °C using a Schlenk line to remove the moisture before the diluted 1-dodecanethiol solution was kept at 50 °C under an argon atmosphere. In a typical reaction, the pristine CsPbBr_3_ NCs dispersed in hexane (2 mL) with oleic acid (OA) ligands were transferred into a sealed and argon-purged 4 mL chromatographic bottle, into which 0.1 mL dried DDT–hexane solution was injected. The mixture was sonicated at 50 °C for 2 h for ligand exchange.

### Pump–probe experiment

The femtosecond pump–probe TA measurements were performed using a regenerative amplified Ti:sapphire laser system (Coherent; 800 nm, 70 fs, 6 mJ per pulse, and 1 kHz repetition rate) as the laser source and a Femto-100 spectrometer (Time-Tech LLC) as the spectrometer. Briefly, the 800 nm output pulse from the regenerative amplifier was split in two parts with a 50% beam splitter. The transmitted part was used to pump a TOPAS Optical Parametric Amplifier (OPA) which generated a wavelength-tunable laser pulse from 250 nm to 2.5 μm as the pump beam. The reflected 800 nm beam was split again into two parts. One part with less than 10% was attenuated with a neutral density filter and focused into a 2 mm thick sapphire or CaF_2_ window to generate a white light continuum (WLC) used as the probe beam. The probe beam was focused with an Al parabolic reflector onto the sample. After the sample, the probe beam was collimated and then focused into a fiber-coupled spectrometer with CMOS sensors and detected at a frequency of 1 kHz. The intensity of the pump pulse used in the experiment was controlled using a variable neutral-density filter wheel. The delay between the pump and probe pulses was controlled using a motorized delay stage. The pump pulses were chopped using a synchronized chopper at 500 Hz and the absorbance change was calculated with two adjacent probe pulses (pump-blocked and pump-unblocked). The samples were placed in 1 mm cuvettes and were vigorously stirred in all the measurements.

## Conflicts of interest

There are no conflicts to declare.

## Supplementary Material

Supplementary information
