# Supplementary material for: On the absence of a phonon bottleneck in strongly confined CsPbBr3 perovskite nanocrystals
Source: Chem Sci. 2019 May 6;10(23):5983–9. doi: 10.1039/c9sc01339c (PMC6566378; doi:10.1039/c9sc01339c)
Supplement: Supplementary file 1 [file SC-010-C9SC01339C-s001.pdf]

Electronic Supplementary Material (ESI) for Chemical Science.
This journal is © The Royal Society of Chemistry 2019

Electronic Supplementary Information for:

On the absence of a phonon bottleneck in strongly-confined $CsPbBr_3$ perovskite nanocrystals

Yulu Li,[1,2] Runchen Lai,[1] Xiao Luo,[1] Xue Liu,[1] Tao Ding,[1] Xin Lu[2] and Kaifeng Wu[1]*

[1]State Key Laboratory of Molecular Reaction Dynamics and Dynamics Research Center for Energy and Environmental Materials, Dalian Institute of Chemical Physics, Chinese Academy of Sciences, Dalian, Liaoning 116023,China

[2]Departmental of Chemistry, College of Chemistry and Chemical Engineering, Xiamen University, Xiamen, Fujian 361005, China

* Corresponding Author: kwu@dicp.ac.cn

Content list:

Figures S1-S13

Wavefunction calculations

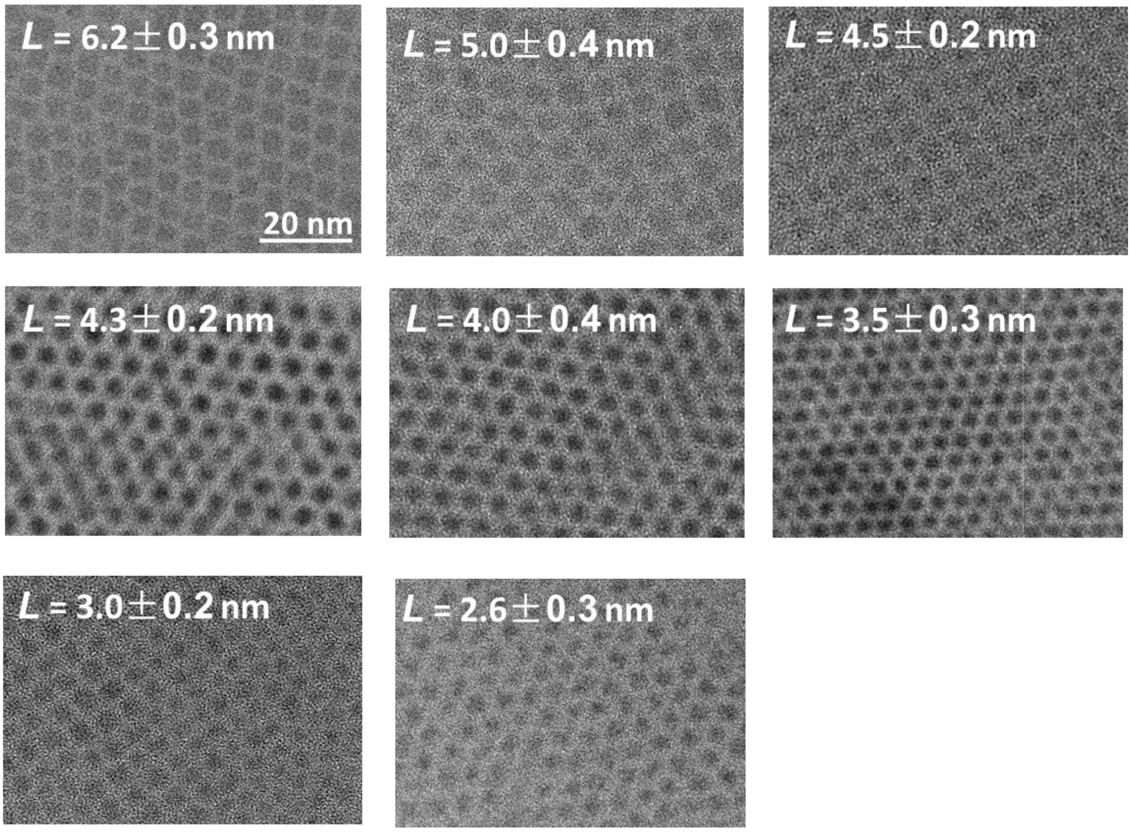

Figure S1. TEM images of $CsPbBr_3$ NCs with varying sizes from 2.6 to 6.2 nm.

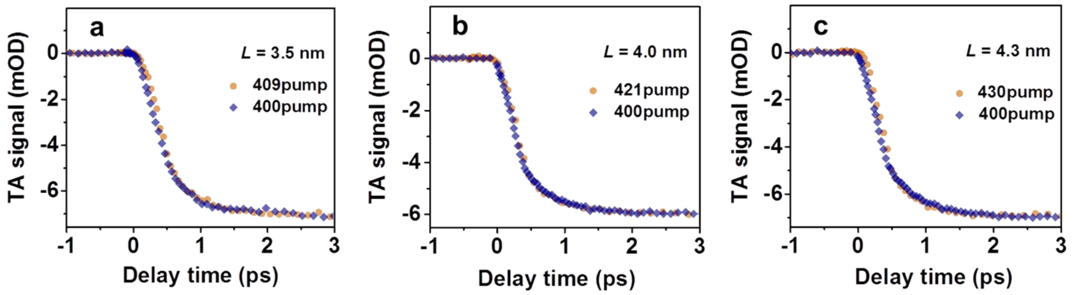

Figure S2. Hot exciton relaxation dynamics (monitored at B1) for NCs with (a) L = 3.5 nm, (b)L = 4.0 nm and (c) L = 4.3 nm pumped with 400 nm (blue diamonds) and with the indicated wavelengths which selectively excite theirX_2 (orange circles).

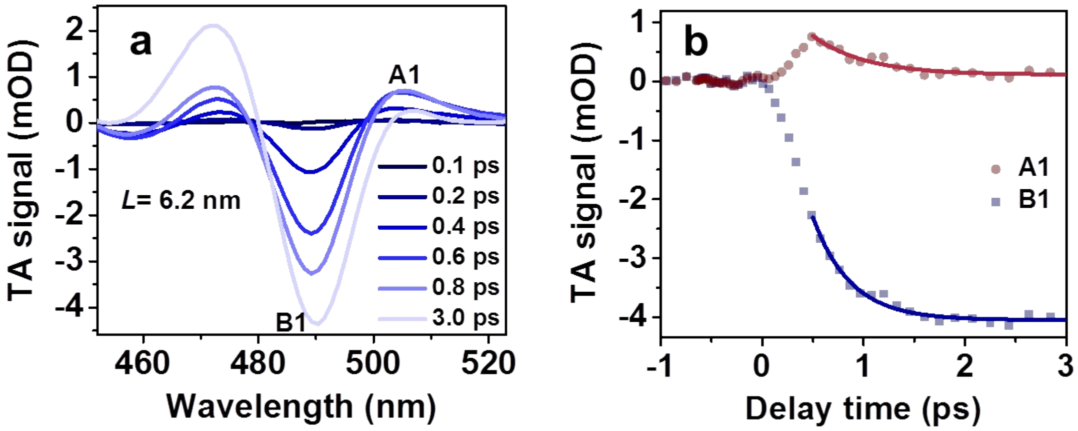

Figure S3. (a) Transient absorption (TA) spectra of L = 6.2 nm NCs at indicated delays following the pump pulse. The bleach feature B1 and absorptive feature A1 are labeled. (b) TA kinetics probed at the peaks of A1 (red circles) and B1 (blue diamonds) are their single-exponential fits (solid lines).

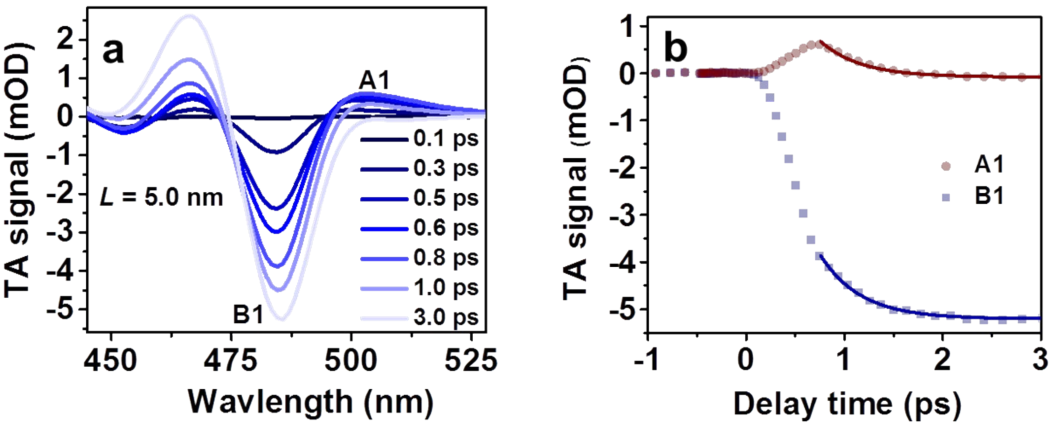

Figure S4. (a) Transient absorption (TA) spectra of L = 5.0 nm NCs at indicated delays following the pump pulse. The bleach feature B1 and absorptive feature A1 are labeled. (b) TA kinetics probed at the peaks of A1 (red circles) and B1 (blue diamonds) are their single-exponential fits (solid lines).

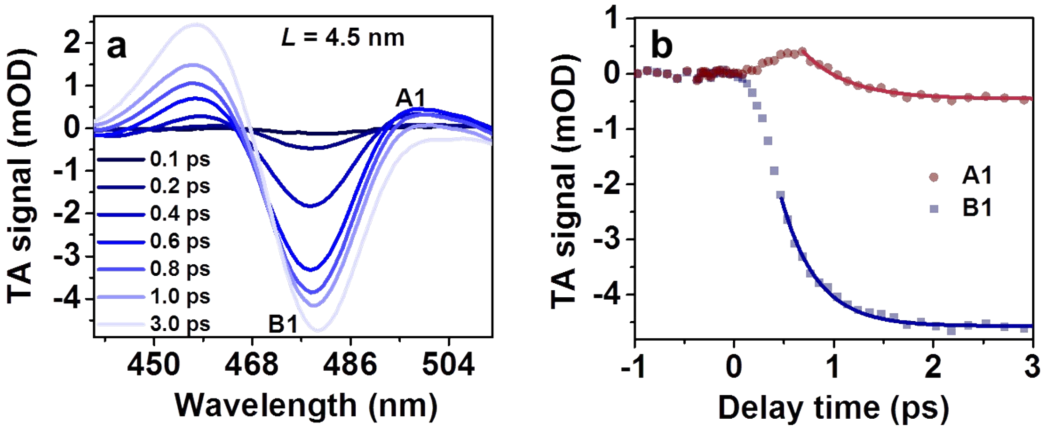

Figure S5. (a) Transient absorption (TA) spectra of L = 4.5 nm NCs at indicated delays following the pump pulse. The bleach feature B1 and absorptive feature A1 are labeled. (b) TA kinetics probed at the peaks of A1 (red circles) and B1 (blue diamonds) are their single-exponential fits (solid lines).

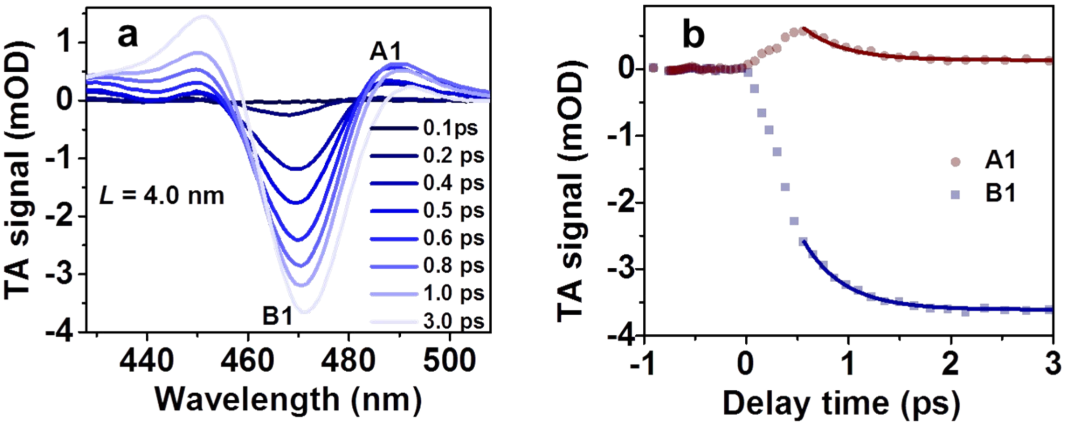

Figure S6. (a) Transient absorption (TA) spectra of L = 4.0 nm NCs at indicated delays following the pump pulse. The bleach feature B1 and absorptive feature A1 are labeled. (b) TA kinetics probed at the peaks of A1 (red circles) and B1 (blue diamonds) are their single-exponential fits (solid lines).

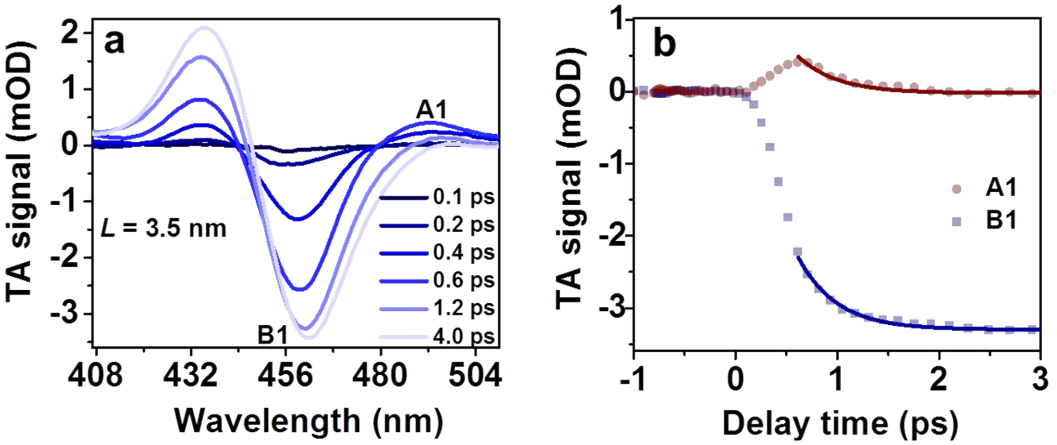

Figure S7. (a) Transient absorption (TA) spectra of L = 3.5 nm NCs at indicated delays following the pump pulse. The bleach feature B1 and absorptive feature A1 are labeled. (b) TA kinetics probed at the peaks of A1 (red circles) and B1 (blue diamonds) are their single-exponential fits (solid lines).

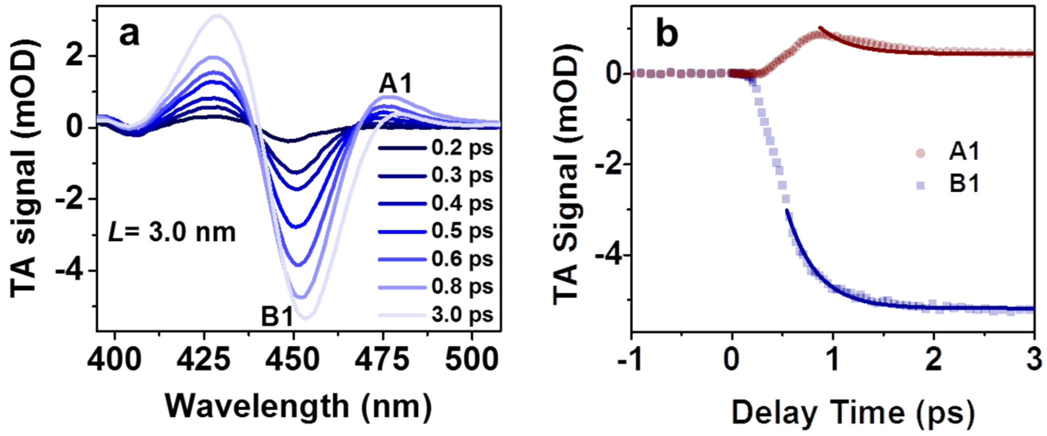

Figure S8. (a) Transient absorption (TA) spectra of L = 3.0 nm NCs at indicated delays following the pump pulse. The bleach feature B1 and absorptive feature A1 are labeled. (b) TA kinetics probed at the peaks of A1 (red circles) and B1 (blue diamonds) are their single-exponential fits (solid lines).

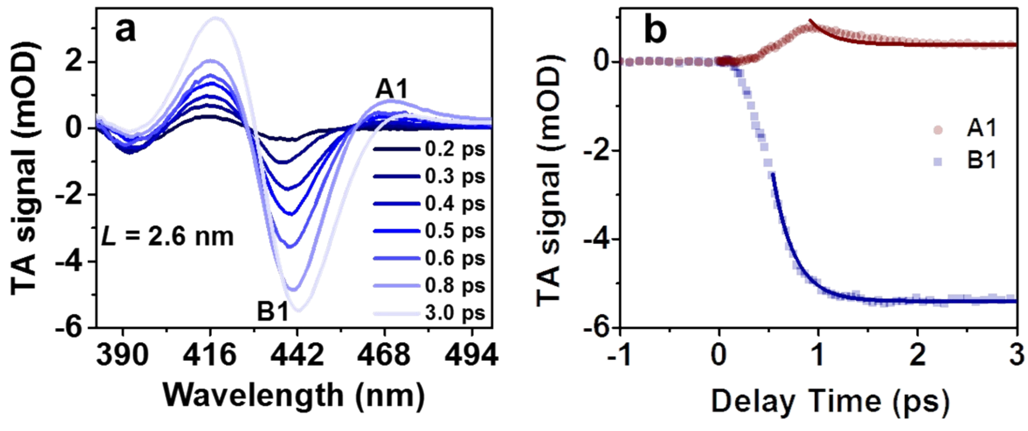

Figure S9. (a) Transient absorption (TA) spectra of L = 2.6 nm NCs at indicated delays following the pump pulse. The bleach feature B1 and absorptive feature A1 are labeled. (b)

TA kinetics probed at the peaks of A1 (red circles) and B1 (blue diamonds) are their single-exponential fits (solid lines).

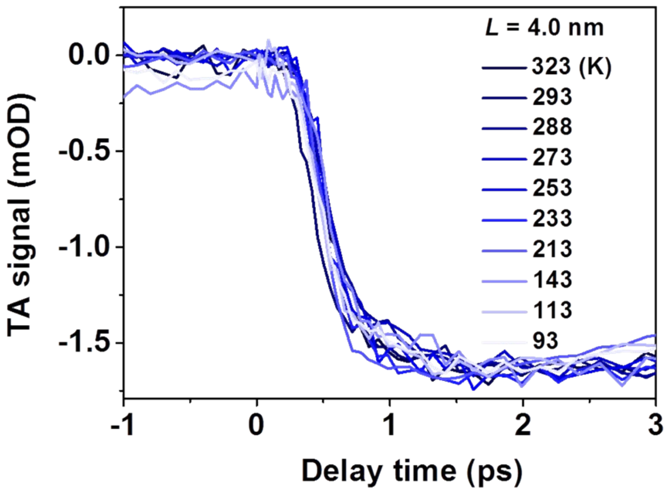

Figure S10. Hot exciton relaxation dynamics (monitored at B1) for L = 4.0 nm NCs at varying temperatures.

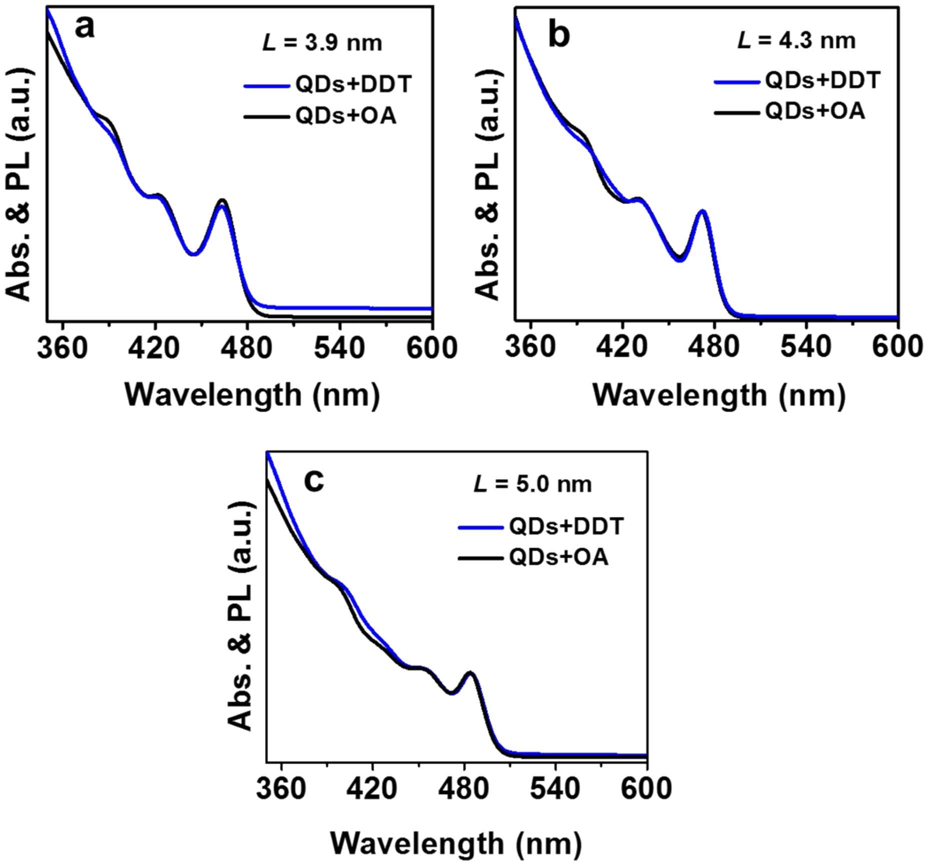

Figure S11. Absorption spectra of pristine NCs (with OA ligands; black solid lines) and DDT-capped NCs (sky blue solid lines) of samples with (a) L = 3.9 nm, (b) L = 4.3 nm, and (c) L = 5.0 nm.

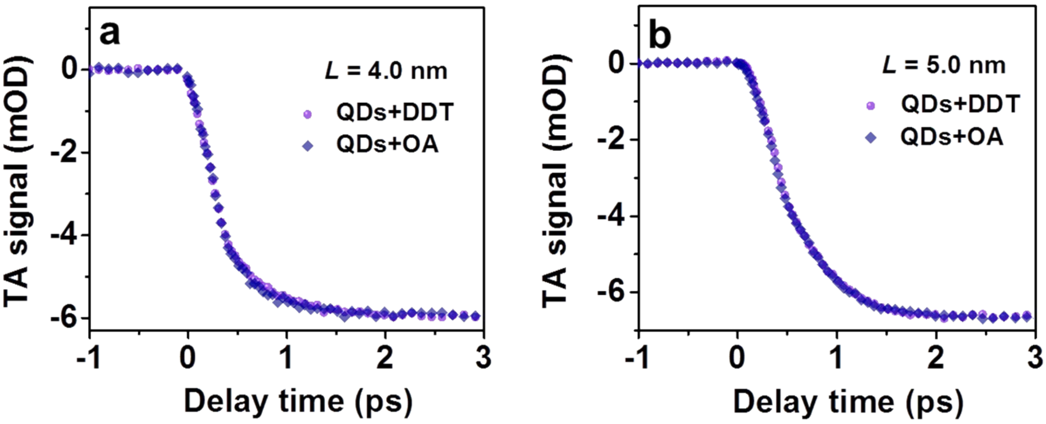

Figure S12. Hot exciton relaxation dynamics (monitored at B1) for (a) L= 4.0 nm, (b) L= 5.0 nm pristine NCs (with OA ligands; blue diamonds) and DDT-capped NCs (purple circles).

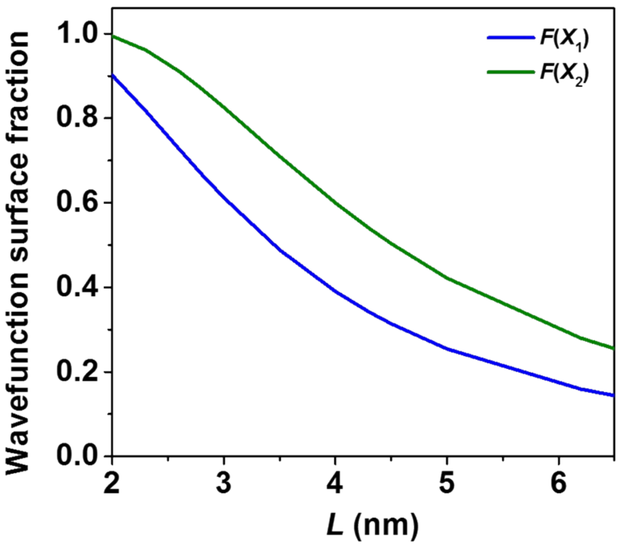

Figure S13. Calculated surface fraction of wavefunctions for X_2 (green) and X_1 (blue).

Wavefunction calculations

Exciton wavefunctions of $CsPbBr_3$NCs were calculated by a single-band effective mass approximation model (EMA) in COMSOL Multiphysics® Modeling Software using the semiconductor module. Note that because of similar electron and hole effective masses in $CsPbBr_3$, calculating either one should be a representation for exciton wavefunction. Here we calculate wavefunctions for the first and second hole states to represent X_1 and X_2. We considered the hole as a particle in 2-region cubic potentials, whose sizes were defined by the size of the cubic $CsPbBr_3$NCs (measured by TEM) and the overall size of the NC-ligand complexes, respectively. The length of the ligand chain was assumed as 2 nm. The potentials were set as follows:

$$V(x)=\begin{cases}0, & -L/2\le x\le L/2\\ V_{OA-HOMO}, & -L/2-2\le x\le -L/2 \ and \ L/2\le x\le L/2+2\end{cases} \quad (S1)$$

in which L stands for the edge length of the cubic $CsPbBr_3$NCs in the unit of nm, and $V_{OA-HOMO}$ is the energy difference of the HOMO of oleic acid molecules and the top of the valence band in the bulk $CsPbBr_3$ material.

The effective masses of the holes in the above two regions were defined as:

$$m_h^*(x)=\begin{cases}m_{h,CPB}^*, & -L/2\le x\le L/2\\ 0.45m_e, & -L/2-2\le x\le -L/2 \ and \ L/2-2\le x\le L/2+2\end{cases} \quad (S2)$$

in which m_e is the mass of free electron and $m_{h,CPB}^*$ the hole effective mass in $CsPbBr_3$ bulk. The detailed values of the above parameters were listed below.

	$V_{OA-HOMO}$	$m_{h,CPB}^*$
Values	0.83 eV[1]	$0.22m_e$[2]

Following the procedures in ref[3], we obtain the surface fraction (F) of wavefunction interacting with ligands by integrating wavefunctions in the outermost unit cells and tunneling into NC surfaces, i.e., in the range of L/2-0.6 to L/2+2 (in the unit of nm). 0.6 nm is the unit cell parameter for $CsPbBr_3$ lattice. The surface fractions calculated for X_1 and X_2 are plotted in Fig. S13.

References for SI:

1. D. Valencia, I. García-Cruz, V. H. Uc, L. F. Ramírez-Verduzco, M. A. Amezcua-Allieri, J. J. B. Aburto, *Biomass and Bioenergy*, 2018, 112, 37-44.
2. K. Miyata, D. Meggiolaro, M. T. Trinh, P. P. Joshi, E. Mosconi, S. C. Jones, F. De Angelis and X.-Y. Zhu, *Sci. Adv.*, 2017, 3, e1701217.
3. R. R. Cooney, S. L. Sewall, E. A. Dias, D. M. Sagar, K. E. H. Anderson and P. Kambhampati, *Phys. Rev. B*, 2007, 75, 245311.
